# Comparison of Cross-Sectional Area of Pubovisceral Muscle in Nulliparous and Primiparous Women

**DOI:** 10.1007/s00192-024-05733-3

**Published:** 2024-02-20

**Authors:** Alexandra Regendova, Adela Samesova, Katerina Zapletalova, Sabina Horejskova, Zuzana Svata, Monika Hrdouskova, Jan Zapletal, Ladislav Krofta, Lucie Hajkova Hympanova

**Affiliations:** 1 Charles University, Third Faculty of Medicine, Institute for the Care of Mother and Child, Prague, Czech Republic; 2 Department of Development and Regeneration, Cluster Woman and Child, Biomedical Sciences, KU Leuven, Leuven, Belgium; 3 Klinika JL-MR, Prague, Czech Republic; 4 Department of Obstetrics and Gynaecology, Third Faculty of Medicine, Charles University and University Hospital Kralovske Vinohrady, Prague, Czech Republic

**Keywords:** Magnetic resonance imaging, Ultrasound, Pubovisceral muscle, Cross-sectional area, Denervation

## Abstract

**Introduction and Hypothesis:**

The main risk factor for pelvic floor disorders is vaginal delivery, which may cause levator ani muscle (LAM) injury and denervation. LAM includes pubovisceral muscle (PVM, pubococcygeus), puborectalis muscle (PRM), and iliococcygeus muscle. We hypothesize that primiparous women with low pelvic floor muscle contraction have a reduced PVM cross-sectional area (CSA) compared to nulliparous women.

**Methods (Sample Size and Statistical Approaches):**

This single-centre prospective observational study compared healthy nulliparous (*n* = 40) to primiparous (*n* = 40) women after vaginal delivery without LAM avulsion and Oxford score ≤ 3. Demographics, questionnaires (ICIQ-UI-SF, OAB-Q-SF, PISQ-12), POP-Q, Oxford score, ultrasound measurements (minimal anteroposterior and lateral diameters, hiatal area, PRM thickness, levator-urethra gap) and magnetic resonance imaging (MRI)—PVM CSA were evaluated. Normality was tested, and an appropriate test was used to compare the groups. Power calculation suggested 40 participants per group.

**Results:**

The primiparous group was older, had a higher BMI, and their hiatal area on ultrasound at contraction was larger compared to the nulliparous group. The CSA of the left-sided PVM (1.15 ± 0.50 cm^2^) was larger compared to the right side (1.03 ± 0.50 cm^2^), *p* = 0.02 in nulliparous women. The PVM CSA of primiparous women with low Oxford score was reduced compared to nulliparous (0.87 ± 0.30 versus 1.09 ± 0.50 cm^2^, *p* = 0.006). The intra-rater reliability for PVM CSA had an ICC of 0.90 and inter-rater ICC of 0.77.

**Conclusions:**

Primiparous women after vaginal delivery with low pelvic floor contraction force had reduced PVM CSA on MRI images compared to nulliparous women.

## Introduction

Pelvic floor disorders (PFDs), including pelvic organ prolapse, urinary and fecal incontinence decrease the quality of life of every fourth woman [1]. The main known risk factor for PFDs is vaginal delivery [2, 3] causing pelvic floor muscle avulsion, ischemia, or denervation [4, 5]. The LAM avulsion (detachment from its origin at the pubis) has been described in 2–36% women after vaginal delivery [6]. The muscle overstretching during delivery can cause denervation, which can lead to muscular atrophy [7, 8]. The electrical stimulation tests of pudendal nerve function and external anal sphincter tone revealed that the trauma of a prolonged second stage, forceps delivery, third-degree perineal tear, or delivery of extensively large child may be associated with permanent damage of nerves and perineal structures in approximately 40% of cases [9].

Based on origin-insertion anatomical terminology, the levator ani muscle (LAM) has these parts: 1/ iliococcygeus, 2/ puborectalis (PRM) and 3/ pubovisceral muscle (PVM, also known as pubococcygeus). The pubovisceral part has three subdivisions: puboperinealis, pubovaginalis, and puboanalis [10]. In this article, we will follow this terminology and for details please see reference [10].

Ultrasound (US) and magnetic resonance imaging (MRI) are frequently used to investigate changes in pelvic floor muscles. However, recently described techniques do not allow us to measure comparable parts of LAM by US and MRI. US measurements within this study were performed to add additional participant characteristic, evaluate avulsion and contraction. The recent ultrasound technique is not able to distinguish LAM subdivisions at level III. The PVM and PRM both origin at the pubis but have different lines of action [11]. The commonly used ultrasound scans visualize all subdivisions which originate from pubis, including PVM and PRM, and further follows mainly the line of action of PRM. The MRI is more detailed, on the other hand the US is much more available. Within this study, we aimed to measure a precisely defined part of the LAM. This was previously described by the group of DeLancey, who published the measurement of PVM cross-sectional area (CSA) [12] and showed a good reproducibility. Therefore, we followed this methodology. To our knowledge, the comparison between a group of nulliparous women without PFDs with a group of women after first vaginal delivery with low pelvic floor muscle contraction has not been described yet. Additionally, the possibility to pool the data together with other small-size MRI studies may give us a better idea about the population variability.

The primary hypothesis was that women after the first vaginal delivery with low contraction force have reduced PVM CSA compared to nulliparous women.

## Materials and Methods

### Study Design and Participant’s Characteristics

This single-centre prospective observational study has been approved by the Ethics Committee of the Institute for the Care of Mother and Child and Institutional review board (Prague, Czech Republic, n.2021/28_1/2).

Two groups of women of reproductive age were included 1/ nulliparous and 2/ primiparous after their first vaginal delivery (Table 1). Women with a medical history of gynaecological surgery or disorder with possible impact on the pelvic floor (i.e. urological, intestinal, neurological) were excluded. Additional exclusion criteria for the primiparous group were: 1/ assisted vaginal delivery (forceps, vacuum extraction), 2/ labour induction, 3/pregnancy-related disorders, 4/ perineal tear grade III and higher, 5/ suspicion of LAM avulsion by ultrasound [13] or palpation [14] 6/ Oxford score 4 and 5 [15].

**Table 1:** Inclusion and exclusion criteria for recruitment of volunteers

Inclusion criteria		Exclusion criteria
Nulliparous women	Reproductive age	history of gynecological surgery or disorder with possible impact on pelvic floor
Primiparous women	Reproductive age vaginal birth	history of gynecological surgery or disorder with possible impact on pelvic floor assisted vaginal delivery (forceps, vacuum extraction) labour induction pregnancy-related disorders perineal tear grade III-IV (women with episiotomy were included) suspicion of LAM avulsion by ultrasound or palpation Oxford score 4 or 5 after delivery

LAM levator ani muscle

Participants signed informed consent and completed standardized pelvic floor disorder-related questionnaires: the Incontinence Questionnaire-Urinary Incontinence-Short Form (ICIQ-UI-SF), Overactive Bladder-Questionnaire short form (OAB-Q-SF), A short form of the Pelvic Organ Prolapse/Urinary Incontinence Sexual Questionnaire (PISQ-12). We recorded their characteristics (age, BMI, race, parity, delivery type, medical history) and information about pelvic floor muscle training (yes or no).

The nulliparous group was recruited at a non-urogynecological outpatient clinic. The clinical examination and ultrasound were performed as soon as possible after their recruitment for the study and MRI a few months afterward. The primiparous group was recruited at the delivery ward and at the postpartum care department. In case of willingness to participate, women were examined clinically and by ultrasound 6 weeks postpartum. Women with recognized avulsion (ultrasound, palpation) or strong pelvic floor muscle contraction (Oxford score) were excluded. The MRI was performed as soon as possible after the ultrasound examination, within 3–6 months postpartum.

### Clinical Examination

The stage of prolapse was quantified by an approved objective system for describing, quantitating and staging pelvic support in women, the Pelvic Organ Prolapse Quantification System (POP-Q) [16]. The Oxford scale was used to characterize pelvic floor muscle contraction by manual palpation [15].

### Ultrasound Scanning and Evaluation

Ultrasound scans were obtained with a GE Voluson E10 system (General Electric Healthcare, Chicago, IL) by abdominal curved array volume transducers (4–8 MHz) (RM6C H48671ZG). 3D/4D ultrasound acquisition of the pelvic floor was acquired by transperineal approach with the following settings: a field of view ≥ 70° and an acquisition angle ≥ 85°, as previously described [17, 18]. The patient was in the dorsal lithotomy position with an empty bladder and the probe was placed vertically on the central perineum, providing a mid-sagittal view.

**Fig. 1:**
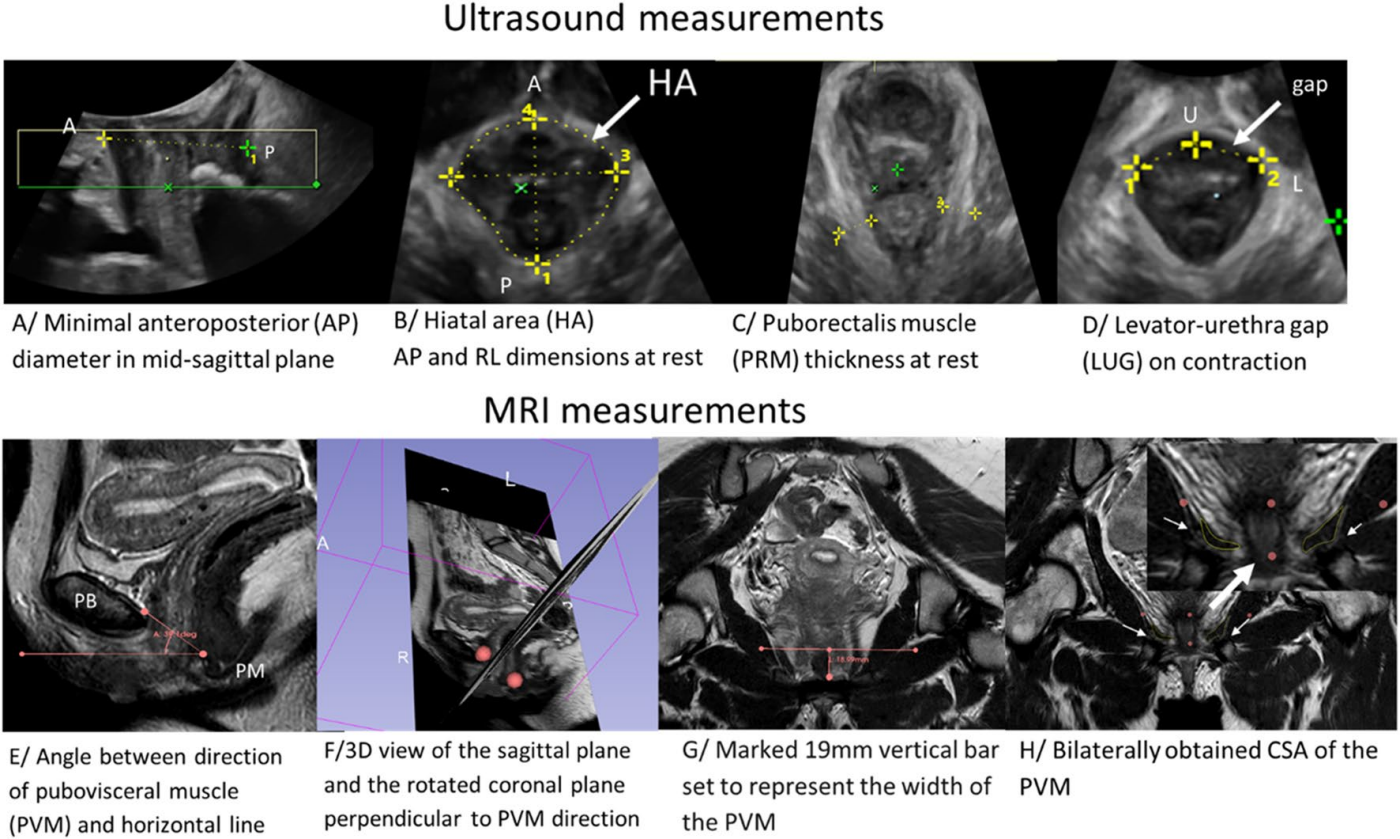
The representative figures of ultrasound (US) and magnetic resonance imaging (MRI) measurements. Figure **A**–**D** represents measured ultrasound dimensions. Figure **E**–**H** represents individual steps of one MRI measurement. The Figure H shows visible difference in left and right sided pubovisceral muscle cross sectional area on MRI. In the right upper corner of the Figure **H** is larger magnification. Abbreviations: A – anterior, P – posterior, HA – hiatal area, U – urethra, L – levator ani muscle, PB – pubic bone, PM—perineal membrane, PRM – puborectalis muscle, PVM—pubovisceral muscle

4D volumes were acquired at rest, during the Valsalva maneuver and on maximal pelvic floor muscle contraction. The Valsalva maneuver as well as contraction were explained and rehearsed with each patient prior to scanning. The US postprocessing was performed in the 4D View v 2.1–5.0 software (GE Medical Systems) by a single investigator blinded to all clinical data. The following parameters were measured for this study. The plane of minimal anteroposterior (AP) diameter was identified in the mid-sagittal image (Fig. 1A). In the axial plane at this level, the AP and lateral diameters of the levator hiatus as well as hiatal area were determined and measured from rendered volumes at rest, on contraction and during maximal Valsalva (Fig. 1B). Levator muscle thickness was measured in the plane of maximal muscle thickness at rest (Fig. 1C). Determined by slow moving of the plane from minimal hiatal dimensions cranially until the maximal diameter was reached [19]. The levator-urethra gap (LUG) was defined using tomographic ultrasound imaging (TUI) of volume data obtained on maximal contraction (Fig. 1D). For LUG measurements, callipers were placed in the centre of the hypoechogenic structure indicating urethral mucosa and on the most medial aspect of the muscle insertion on the pubic ramus [19, 20].

### MRI Scanning and Evaluation

The MRI scans were acquired in the supine position by 3 T Philips Ingenia (Philips Healthcare, Best, the Netherlands) with an additional dStream Torso coil over the pelvis. MRI included coronal, axial, and sagittal proton density-weighted sequences (TR/TE, 3000- 5000/82 ms), slice thickness 2 mm, gap 0 mm. Scans were acquired at rest. Women were asked to empty their bladder before scanning, any other additional preparation (i.e. bowl) was not performed. The MRI scans were evaluated offline by two investigators blinded to all clinical data. The 3D Slicer (v. 5.2.2) and ImageJ software (1.52j, NIH, USA) were used for this purpose.

The PVM CSA was measured as previously described by Masteling et al. [12]. Briefly, the MR images were imported into 3D Slicer, where slices containing the volume of interest were identified in a plane perpendicular to the muscle direction. To identify a plane perpendicular to the PVM, the PVM fibres direction was first established between origin and insertion, namely the inner surface of the pubic bone and its insertion into the perineal structures (Fig. 1E). In the coronal plane perpendicular to the muscle direction, the boundaries of the PVM were identified and images of this region were taken. The captured images were then exported into ImageJ for measurement of CSA (Fig. 1H). The CSA of PVM was outlined and the largest CSA was selected.

The MRI measurements were performed by two investigators blinded to group allocation. To confirm repeatability, one investigator performed measurements twice two months apart. For inter-rater repeatability, and for reporting on the measurements, the first measurements were used.

### Statistics

Statistical analysis was performed mainly by using the GraphPad Prism version 7.0 (GraphPad Software, Inc; La Jolla, USA). First, the normality was tested by D'Agostino-Pearson test. For continuous, normally distributed variables the unpaired Student’s t-test was used and Mann–Whitney was used for not normally distributed variables. For the comparison of paired observations, i.e. left and right side of the LAM CSA, inter-and intra-rater reliability paired t-test was used. For comparison of more groups, one-way ANOVA followed by Tukey’s post hoc test was used for normally distributed data. Kruskal–Wallis followed by Dunn’s post-test was used for not normally distributed data. The statistical significance level was defined as *p* < 0.05. All data were reported as mean and standard deviation (± SD).

In an animal experiment the denervated limb muscle lost 40% of weight in one month and up to 80% in 6 months. Other data related to denervation feasible for power calculation have not been found in the literature. We expected a scenario of lower difference, considering partial denervation, reinnervation and population variability. With expectation of 40% difference between groups, power calculation for the MRI primary outcome measurement PVM SCA suggested 40 participants per group [7].

Intraclass correlation coefficients (ICC) (mode: two-way random; type: absolute agreement) (95% confidence interval) were used to assess measurement agreement within a rater over time and between raters. ICC was conducted using SPSS (version 19, IBM, Chicago, IL, USA).

## Results

### Groups Characteristics

The demographic and other characteristics are summarized in Table 2. Women in the primiparous group were older and had higher BMI. In both groups, Caucasian race was dominant. Most of the women were non-smokers. Women in both groups had some additional disorders that do not affect their pelvic floor condition. The ICIQ-UI-SF score was significantly higher in the primiparous group, the OAB-Q-SF and PISQ-12 did not significantly differ between groups.

**Table 2:** The table includes demographics and other characteristics of the studied population

Demographic and other characteristics				
		Nulliparous (*n* = 40)	Primiparous (*n* = 40)	*p*
Age (mean ± SD) years		27.85 ± 3.86	30.28 ± 3.47	0.002
Race (%)	Caucasian	39 (97.5%)	39 (97.5%)	NA
	Asian	1 (2.5%)	1 (2.5%)	NA
BMI (mean ± SD)		22.68 ± 4.84	24.28 ± 3.92	0.02
Smoking (%)	Never smokers	36 (90%)	40 (100%)	NA
	Current smokers	4 (10%)	0 (0%)	NA
	Quitter	0 (0%)	0 (0%)	NA
Disorders (%)	Thyreopathy	2 (5%)	2 (5%)	NA
	History of lower UTI	3 (7.5%)	3 (7.5%)	
	Arterial hypertension	2 (5%)	0 (0%)	
	Rheumatoid arthritis	1 (2.5%)	0 (0%)	
	Polycystic ovary syndrome	2 (5%)	1 (2.5%)	
PFMT (pelvic floor muscle training)		8 (20%)	12 (30%)	NA
Newborn birth weight (g)		NA	3471.0 ± 368.3	NA
ICIQ-UI-SF score (0–21) [95%CI]		0.83 ± 1.93 [0.21 1.44]	2.80 ± 4.27 [1.43 4.17]	0.02
OAB-Q-SF		24.50 ± 10.07 [21.28 27.72]	26.55 ± 9.85 [23.40 29.70]	0.20
PISQ-12		40.70 ± 2.44 [39.92 41.48]	39.14 ± 4.39 [37.67 40.60]	0.055
Oxford score		3.07 ± 1.55	1.40 ± 1.07	< 0.0001
POP-Q:				
Aa		-2.8 ± 0.4 [-3.0 -2.7]	-1.9 ± 0.8 [-2.2 -1.6]	0.0001
Ba		-2.8 ± 0.4 [-3.0 -2.7]	-1.9 ± 0.8 [-2.1 -1.6]	0.0001
C		-7.4 ± 2.6 [-8.3 -6.6]	-6.2 ± 2.9 [-7.2 -5.3]	0.005
D		-8.0 ± 4.7 [-9.5 -6.5]	-7.4 ± 4.0 [-8.7 -6.1]	0.03
Ap		-2.9 ± 0.5 [-3.0 -2.7]	-2.2 ± 0.7 [-2.5 -2.0]	0.0001
Bp		-2.7 ± 1.1 [-3.0 -2.3]	-2.0 ± 1.1 [-2.4 -1.7]	0.0001
gh		2.6 ± 0.9 [-2.3 -2.9]	3.6 ± 0.9 [-2.7 -3.3]	0.08
pb		-3.0 ± 2.7 [2.8 3.2]	-2.8 ± 0.5 [2.6 2.9]	0.07
TVL		9.4 ± 0.8 [9.1 9.7]	8.7 ± 1.4 [8.3 9.2]	0.01
MRI: PVM CSA (cm^2^)		1.09 ± 0.50 [0.98 1.20]	0.87 ± 0.29 [0.80 0.93]	0.006
US: PRM thickness at rest (cm)		0.96 ± 0.21 [0.92 1.01]	0.91 ± 0.20 [0.87 0.96]	ns
US Levator-urethra gap (LUG) on contraction		1.94 ± 0.23 [1.90 2.00]	2.13 ± 0.41 [2.04 2.23]	0.035

The statistical significance level was defined as *p* < 0.05. Results are reported as mean ± SD and 95% confidence interval [95%CI]. The race/ethnicity was self-reported and expressed as a real count of cases and percentages. One participant may have more than one disorder. When left and right-sided measurements were performed, those were merged together. Abbreviations: UTI—urinary tract infections, NA – not applicable, ns – not significant ICIQ-UI-SF—the Incontinence Questionnaire-Urinary Incontinence-Short Form, OAB-Q-SF -Overactive Bladder-Questionnaire short form, PISQ-12—A short form of the Pelvic Organ Prolapse/Urinary Incontinence Sexual Questionnaire, POP-Q – Pelvic Organ Prolapse Quantification System, PVM – Pubovisceral muscle, PRM – puborectalis muscle, MRI – magnetic resonance imaging, US – ultrasound, BMI - body mass index

### Ultrasound

Data are summarized in Tables 3 and 4. In the nulliparous group, the hiatal area at contraction and at rest was smaller compared to the area at Valsalva. The hiatal area on contraction was smaller in the nulliparous group compared to the primiparous group. The puborectalis muscle thickness was comparable between the groups.

**Table 3:** Columns with a statistically significant difference are marked by letters: a, b, c, d, e, f

Results of ultrasound measurements							
	Nulliparous (*n* = 40)			Primiparous (*n* = 40)			*p*
	contraction	rest	Valsalva	contraction	rest	Valsalva	
Midsagittal plane							
AP diameter	3.97 ± 0.50 [3.81 4.13]a,b,c	5.04 ± 0.70 [4.82 5.26]a,d	5.20 ± 0.86 [4.93 5.48]b	4.82 ± 0.64 [4.61 5.02]c,e,f	5.79 ± 0.83 [5.53 6.06]d,e	5.90 ± 1.00 [5.58 6.21]f	0.0001
Axial plane							
Hiatal area	10.66 ± 2.02a,b,c	14.90 ± 3.34a	16.54 ± 3.72b	14.55 ± 4.56c,d,e	17.83 ± 5.36d	20.15 ± 6.73e	0.0001
AP diameter	4.18 ± 0.48a,b,c	5.47 ± 0.76a,d	5.63 ± 0.91b,e	5.12 ± 0.77c,f,g	6.28 ± 0.98d,f	6.45 ± 1.09e,g	0.0001
RL diameter	3.56 ± 0.50a,b	3.87 ± 0.55	4.09 ± 0.51a	4.02 ± 0.77b	4.23 ± 0.61	4.44 ± 0.76	0.0001

The statistical significance level was defined as *p* < 0.05. The minimal anteroposterior (AP) diameter was measured in the mid-sagittal plane and axial plane

**Table 4:** The left and right side results

	Nulliparous (*n* = 40)		Primiparous (*n* = 40)		*p*
	Right	Left	Right	Left	
US: Levator-urethra gap (LUG) at contraction	1.93 ± 0.24	1.97 ± 0.23a	2.05 ± 0.34	2.22 ± 0.46 a	0.006
US: PRM thickness at rest (cm)	0.96 ± 0.22	0.97 ± 0.21	0.94 ± 0.19	0.89 ± 0.21	ns
MRI: PVM CSA(cm^2^)	1.03 ± 0.50a,b	1.15 ± 0.50a,c	0.84 ± 0.29b	0.89 ± 0.28c	0.02

The statistical significance level was defined as *p* < 0.05. Letters a, b, c are used to show the significance between columns. Abbreviations: *US* – ultrasound, *MRI* – magnetic resonance imaging, *PVM* – pubovisceral muscle, *CSA* – cross-sectional area, *PRM* – puborectalis muscle

### Magnetic Resonance Imaging (MRI)

**Fig. 2:**
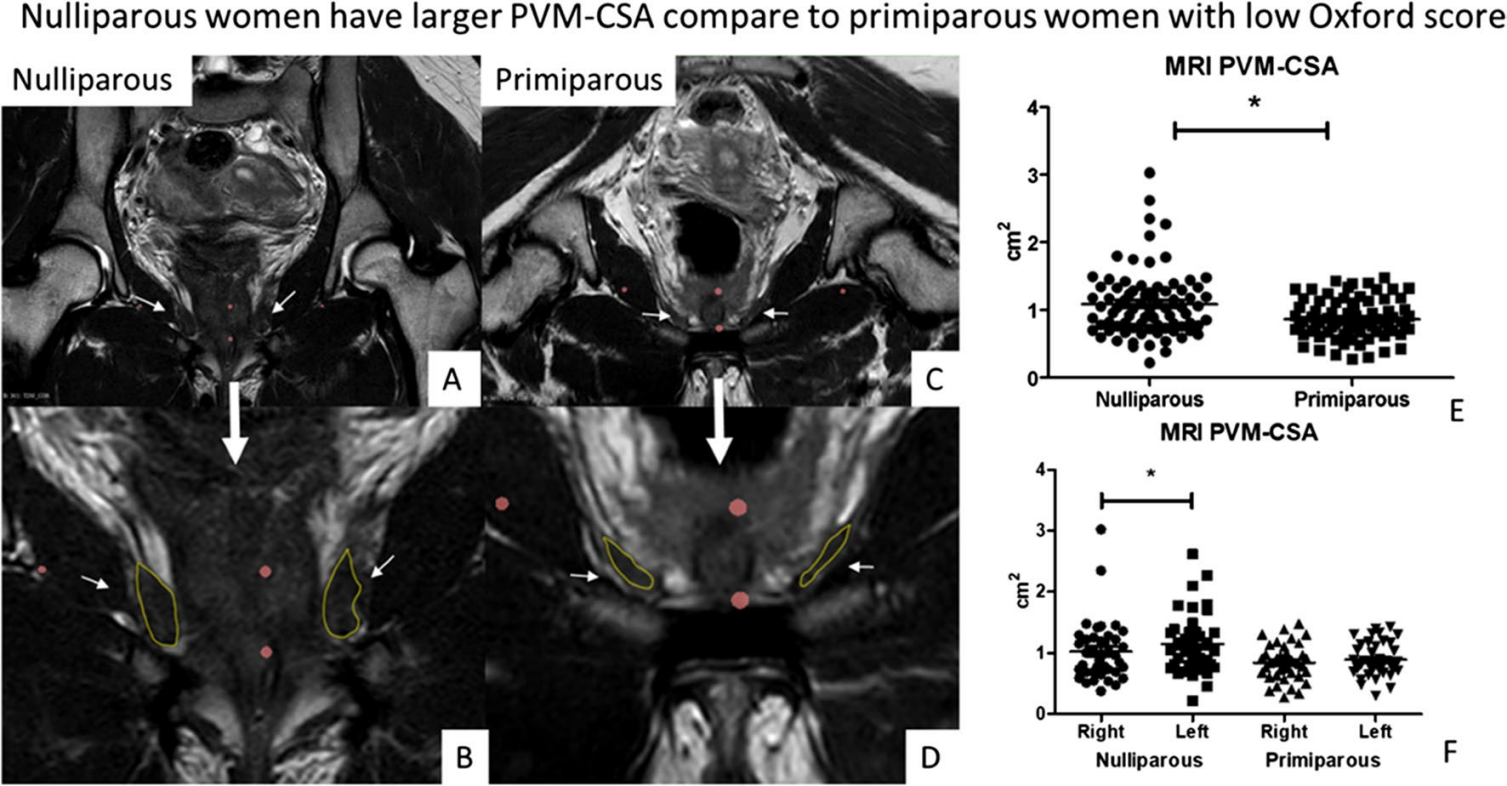
Nulliparous women (**A**, **B**) in our cohort have a larger cross-sectional area compare to primiparous women (**C**, **D**) with low Oxford score visible in graph **E**. The small white arrows point to the pubovisceral muscles. In **B**, **D** are larger magnifications of **A**, **C**. The cross sectional area is marked by a yellow line. In **F** are visible individual values also divided based on the muscle side (left, right). In nulliparous women, the pubovisceral muscle cross-sectional area (PVM CSA) of the left-sided PVM was larger compared to the right side

We were able to analyse the maximum PVM CSA in all cases. In nulliparous women, PVM CSA of the left-sided PVM (1.15 ± 0.50 cm^2^) was larger compared to the right side (1.03 ± 0.50 cm^2^), *p* = 0.02 (Fig. 2F, Table 4). The PVM CSA of primiparous women of 0.87 ± 0.30 was reduced compared to an unselected group of nulliparous women 1.09 ± 0.50 (*p* = 0.006) (Fig. 2E). The primiparous group was also compared to a subgroup of nulliparous with comparably low Oxford scores (*n* = 23; excluded women with values 4, 5). Also, in this case primiparous had reduced PVM CSA 0.87 ± 0.29 compared to the nulliparous 1.06 ± 0.50 (*p* = 0.05).

The intra-rater reliability had an ICC of 0.90 (95% Cl 0.85–0.94), and inter-rater ICC of 0.77 (95% CI 0.56–0.88). 

## Discussion

In our study, primiparous women with intact pelvic floor muscles but low contraction force had approximately 20% reduced pubovisceral muscle cross-sectional area than the nulliparous group. This confirms our initial hypothesis. We have also shown a side difference in PVM CSA of nulliparous women.

The PVM CSA measurements methodology was first published by Masteling et al., who also calculated its confidence intervals for primiparous women after cesarean section [12]. The cross-sectional area of our nulliparous group (1.09 ± 0.50 cm^2^) is similar to their range (1.25 ± 0.29 cm^2^) yet lower. This difference might represent a normal distribution since there were relatively few participants.

Values observed in the primiparous group were lower compared to nulliparous women and also 30% reduced compared to Masteling’s group [12]. Based on our power calculation, we expected a 40% loss of muscular mass if the denervation is complete. We included women with suspected neuronal injury—they delivered vaginally, did not sustain LAM tear, yet presented with a low force during voluntary muscle contraction. Therefore, neuronal damage either was not complete or partially restored since delivery. Several studies support denervation theory while showing an increased pudendal nerve terminal motor latency in 40% of women after vaginal delivery [21], a neuropathic injury of the LAM on electromyography [4], or a reduction in urethral closure pressure [22]. In rat models, urethral sphincter function was restored 14 days after pudendal nerve crushes and vaginal distension [23]. Unfortunately, we could not include neurophysiological testing, which might support our theory on neuronal damage.

Another possible explanation is a change in muscle architecture. During vaginal delivery, the fetal head excessively stretches the LAM, which might lead to micro-traumatization, thinning, elongation and over-stretching. In the postpartum period, the LAM would heal but change its architecture, which might affect the muscle volume.

Contrary to Masteling’s study, the left-sided PVM had a 10% larger cross-section area than its right-side muscle belly [12]. In the human body, muscle asymmetry is frequent and manifests as a preference for the right upper limb [24].

Our intra-rater reliability of PVM CSA measurement was excellent at 0.90; however, the inter-rater ICC 0.77 was just “very good,” contrary to the previously published work, where the lower intra-rater and inter-rater reliability were both excellent, 0.89 [12].

This study has several limitations. Firstly, we were not able to pair participants in our groups. It might be due to different recruitment places and limited time for recruitment. Nulliparous women were less likely to participate and challenging to attract. Primiparous women had to fulfil strict criteria on their pelvic floor condition. Nevertheless, the ideal would be a longitudinal observational group of women to allow comparison of Oxford score and PVM CSA before and after vaginal delivery. Secondly, we used subjective evaluation of PVM contraction because other objective techniques were not accessible at our clinic.

Another limitation we did not expect at the time of planning was an incoherent anatomical nomenclature among published studies. Initially, we aimed to measure the same part of the LAM by ultrasound and MRI, but it was impossible. In MRI images, the pubovisceral part could be precisely distinguished from the puborectal part of LAM [12], but this is hardly possible on 3D/4D ultrasound. The most frequently used axial plane in ultrasound shows the puborectalis at the plane of minimal anteroposterior diameter [25], yet some authors called it pubovisceralis [18]. On the ultrasound, the LAM size was represented by a measurement of PRM thickness and its area [18], but the precise cross-sectional area was, to our knowledge, measured only on PVM by MRI [12]. Therefore, we emphasize adherence to recent anatomical nomenclature. Our future goal is to measure comparable muscles by both techniques.

The nulliparourous women had no history or connection to urogynecological clinic or pelvic floor disorders; however, their willingness to participate could be caused by increased interest in the pelvic floor and therefore not represent the general population.

This study also has several strengths. We performed a power calculation before the study; it was prospectively designed, and we used several methods to evaluate the pelvic floor. 3 T-MRI images obtained are very detailed and comprehensive and could be used for other anatomical purposes. We have also confirmed that PVM CSA measurement is repeatable.

## Conclusion

After vaginal delivery with the intact levator ani but low voluntary contraction force, primiparous women have a reduced cross-sectional area of the pubovisceral muscle on MRI than healthy nulliparous women. A more extensive study or combined data from multiple studies would describe population variability and help us to form a complete conclusion.

## Data Availability

Data available on request.
